# Cardiolipin content, linoleic acid composition, and tafazzin expression in response to skeletal muscle overload and unload stimuli

**DOI:** 10.1038/s41598-017-02089-1

**Published:** 2017-05-17

**Authors:** Val A. Fajardo, John S. Mikhaeil, Cameron F. Leveille, Caitlin Saint, Paul J. LeBlanc

**Affiliations:** Department of Health Sciences, Centre for Bone and Muscle Health, Brock University, St. Catharines, Ontario Canada

## Abstract

Cardiolipin (CL) is a unique mitochondrial phospholipid that, in skeletal muscle, is enriched with linoleic acid (18:2*n*6). Together, CL content and CL 18:2*n*6 composition are critical determinants of mitochondrial function. Skeletal muscle is comprised of slow and fast fibers that have high and low mitochondrial content, respectively. In response to overloading and unloading stimuli, these muscles undergo a fast-to-slow oxidative fiber type shift and a slow-to-fast glycolytic fiber type shift, respectively, with a concomitant change in mitochondrial content. Here, we examined changes in CL content and CL 18:2*n*6 composition under these conditions along with tafazzin (Taz) protein, which is a transacylase enzyme that generates CL lipids enriched with 18:2*n*6. Our results show that CL content, CL 18:2*n*6 composition, and Taz protein content increased with an overload stimulus in plantaris. Conversely, CL content and CL 18:2n6 composition was reduced with an unloaded stimulus in soleus. Interestingly, Taz protein was increased in the unloaded soleus, suggesting that Taz may provide some form of compensation for decreased CL content and CL 18:2*n*6 composition. Together, this study highlights the dynamic nature of CL and Taz in skeletal muscle, and future studies will examine the physiological significance behind the changes in CL content, CL 18:2*n*6 and Taz.

## Introduction

Cardiolipin (CL) is a unique mitochondrial-specific dimeric phospholipid^1^, that acts as a reliable marker of mitochondrial content^2^. Tissues with high oxidative capacity such as slow-twitch skeletal and cardiac muscle have high CL content ranging from 10–20%^3, 4^; and this is known to be critical for mitochondrial respiration and energy metabolism. Specifically, CL physically interacts with, and in turn, activates a large number of mitochondrial proteins including most, if not all of the inner mitochondrial membrane enzymes, along with cytochrome c and creatine kinase^5, 6^. In fact, CL may be considered as a functional ‘glue’ holding the mitochondrial respiratory complexes together to ensure efficient electron flow and proton transport^7–9^.

Along with CL content, its acyl composition is another major determinant of mitochondrial function and energy metabolism^7^. Although CL’s fatty acid composition varies across tissue types^1^, linoleic acid (18:2*n*6) is the dominate fatty acid bound to CL in cardiac and skeletal muscle^4, 10, 11^. In these tissues, reductions in CL 18:2*n*6 composition can compromise mitochondrial function, as shown with impairments in cytochrome c oxidase activity^12–14^. Moreover, reductions in CL 18:2*n*6 composition and CL content due to mutations in the tafazzin gene (*Taz*) is the primary defect underlying Barth syndrome - a rare congenital myopathy characterized by structural and functional mitochondrial abnormalities, cardio/skeletal myopathy, exercise intolerance and greater reactive oxygen species (ROS) production^7, 11, 15–17^. Accumulating evidence within the literature, suggests that Taz is the primary regulator of CL 18:2*n*6 composition^7^. Specifically, its tranacylase function acts to transfer 18:2*n*6 fatty acids from donor phospholipids such as phosphatidylcholine onto immature forms of CL thereby generating the mature forms of CL, which are enriched with 18:2*n*6^1^.

Skeletal muscle fibers differ in their contractile speed and metabolic capacity, which allows for different tasks to be performed^18^. Postural muscles, such as soleus, resist the downward pull of gravity and exhibit a more tonic activity pattern, and therefore require more slow-twitch oxidative fibers that are fatigue resistant and abundant with mitochondria^18, 19^. Conversely, muscles that exhibit phasic activity patterns, such as plantaris, are required for more explosive movements (ie. sprinting and jumping), are rich with fast glycolytic fibers that produce greater force; but contain less mitochondria and, in turn, are less fatigue resistant^18, 20^. Furthermore, muscle is dynamic and adapts to changes in activity patterns, whereby functionally overloading plantaris muscle via synergist ablation of soleus and gastrocnemius muscles causes plantaris to take on a postural role leading to muscle hypertrophy and a fast-to-slow fiber type switch^21–24^. On the other hand, unloading a predominantly slow-twitch muscle such as soleus induces muscle atrophy and a fiber type switch towards the fast-glycolytic phenotype^25, 26^.

Given the known differences in mitochondrial content between the slow-oxidative and fast-glycolytic fibres^18^, it was of interest to determine whether CL content, CL18:2*n*6 composition, and its primary regulator, tafazzin, would follow the slow-oxidative fibre phenotype. Here, we tested the specific hypotheses that: (1) overloaded plantaris would display an increase in CL content, 18:2*n*6 composition, and Taz protein content, which would correspond with a fast-to-slow fiber type shift; and (2) unloaded soleus would display a decrease in CL content, 18:2*n*6 composition, and Taz protein content, which would correspond with a slow-to-fast fiber type shift.

## Results

### Changes in muscle mass and MHC expression in overloaded and unloaded muscles

**Figure 1:**
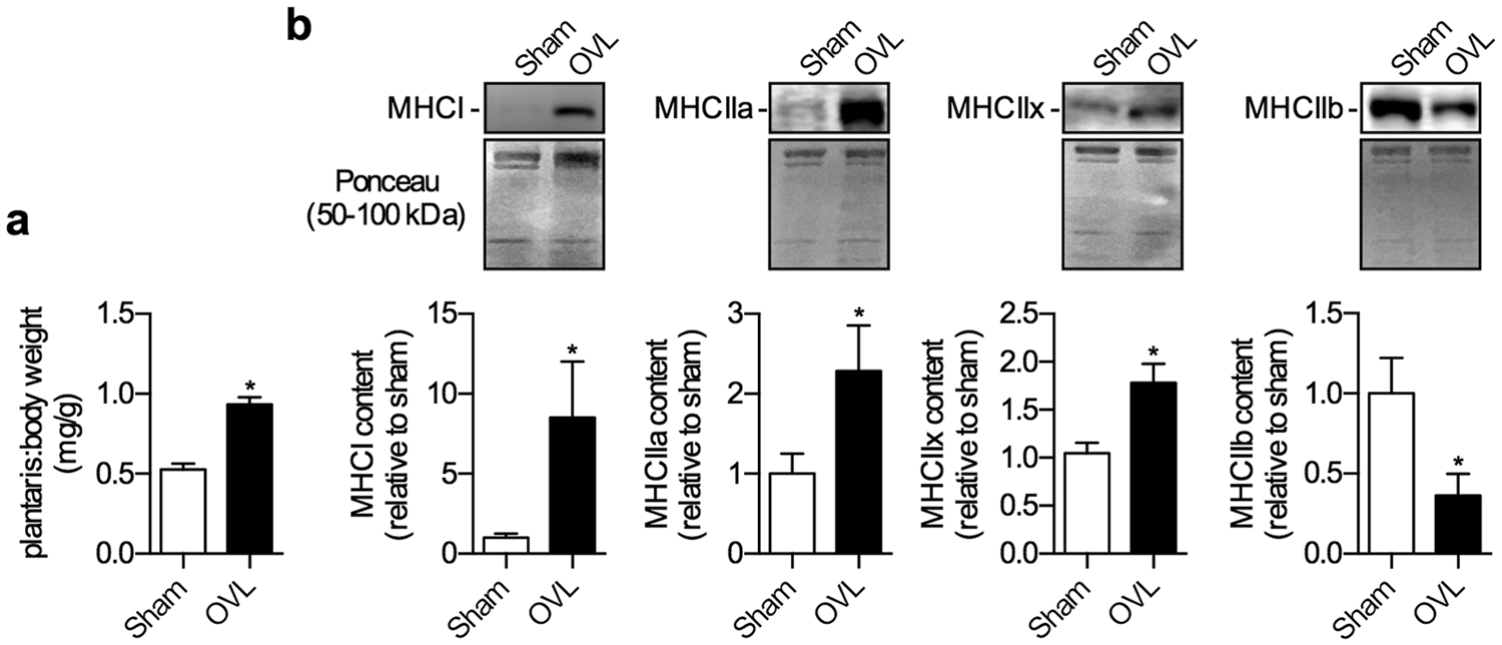
Muscle hypertrophy and a fast-to-slow fiber type shift in the overloaded (OVL) plantaris muscles. (**a**) Plantaris:body weight ratios are significantly higher in the OVL plantaris compared with sham. (**b**) Western blot expression of the MHC isoforms indicates a fast-to-slow fiber type shift with a reduction in IIb and an increase in IIx, IIa, and I. *p ≤ 0.05 using a paired t-test (two-tailed), n = 6 per group. Please refer to Fig. S1 for full length Western blots.

**Figure 2:**
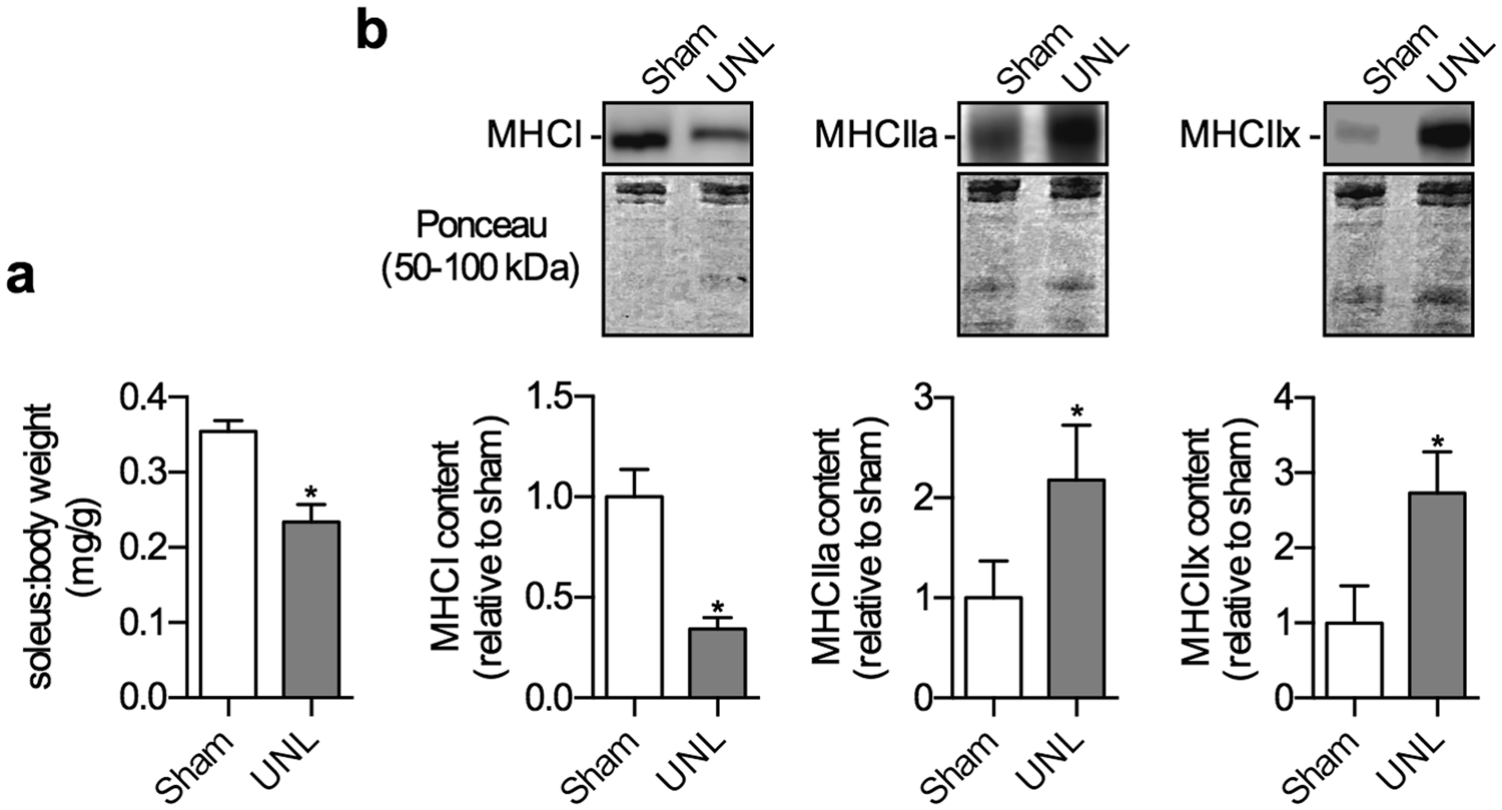
Changes in COXIV protein expression are indicative of changes in mitochondrial content. (**a**) COXIV protein is significantly higher in the overloaded (OVL) plantaris compared to sham. (**b**) COXIV protein is significantly lower in the unloaded (UNL) soleus compared to sham. *p ≤ 0.05 using a paired t-test (one-tailed), n = 6 per group.  Please refer to Fig. S2 for full length Western blots.

As expected, two weeks post-surgery, overloaded plantaris muscles exhibited significant muscle hypertrophy with an approximate 1.8-fold increase in plantaris:body weight ratio compared with sham (Fig. 1a). Examining the absolute muscle weights also reveals a highly significant effect of overloading on plantaris muscle mass (sham, 15.4 ± 1.3 mg vs. OVL, 26.9 ± 0.7 mg; *p* = 0.0004). In addition to the changes in muscle size, overloaded plantaris displayed a shift towards more oxidative fibers with a significant reduction in MHCIIb and a significant increase in MHCI, IIa, and IIx (Fig. 1b; Fig. S1a–d). Conversely, unloaded soleus displayed a 35% reduction in soleus:body weight ratio compared with sham (Fig. 2a). Examining the absolute muscle weights also revealed a highly significant effect of unloading on soleus muscle mass (sham, 10.3 ± 0.4 mg vs. UNL, 6.7 ± 0.6 mg; *p* = 0.006). In addition, these unloaded soleus muscles also exhibited a significant reduction in MHCI expression, and a significant increase in MHCIIa and IIx (Fig. 2b; Fig. S2a–c).

### Cytochrome c oxidase expression as a marker of mitochondrial content

**Figure 3:**
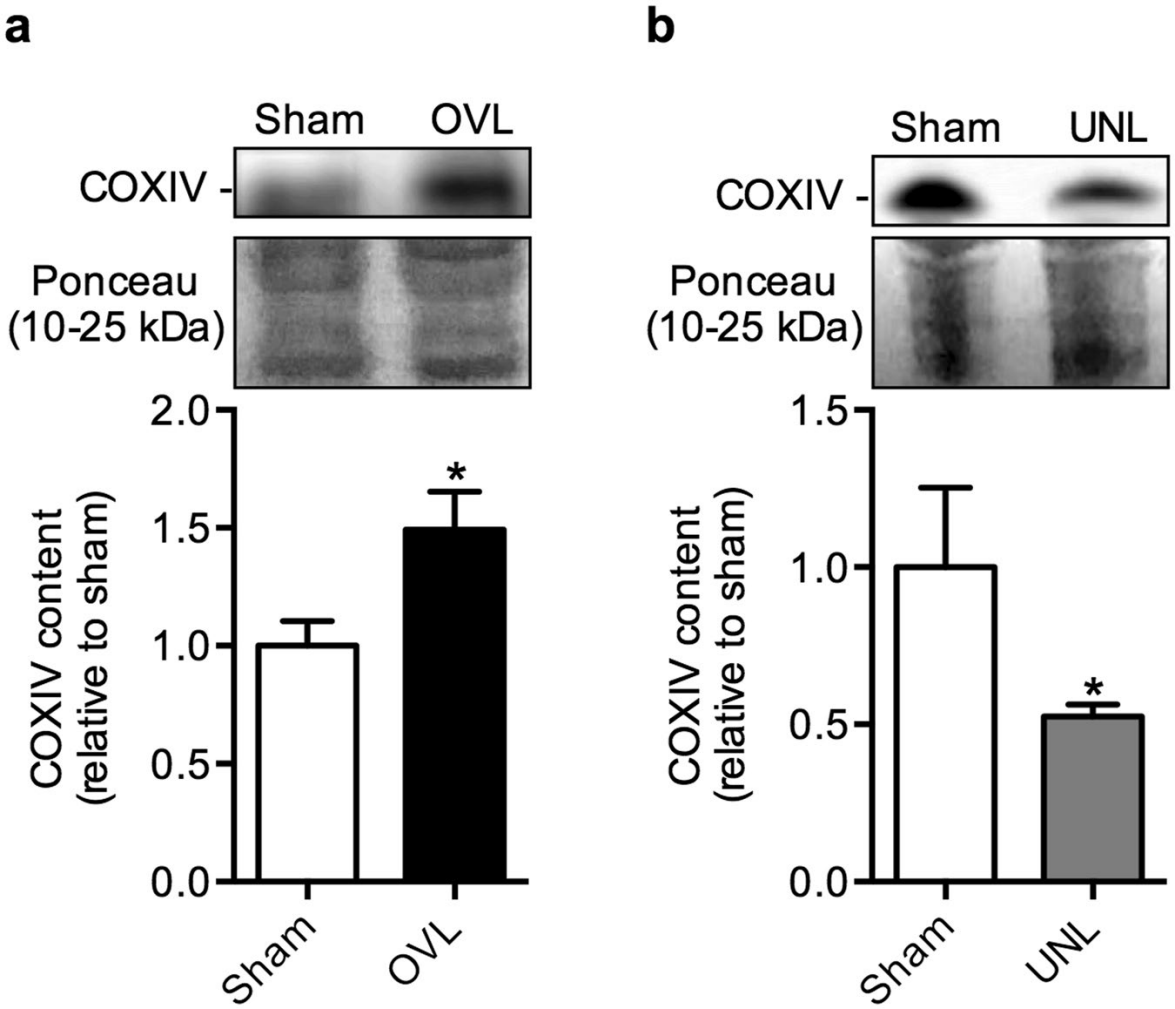
Muscle atrophy and a slow-to-fast fiber type shift in the unloaded (UNL) soleus muscles. (**a**) Soleus:body weight ratios are significantly lower in the UNL soleus compared with sham. (**b**) Western blot expression of the MHC isoforms indicates a slow-to-fast fiber type shift with a reduction in I and an increase in IIa and IIx. *p ≤ 0.05 using a paired t-test (two-tailed), n = 6 per group.  Please refer to Fig. S3 for full length Western blots.

To provide indication of changes in mitochondrial content, we examined the expression of cytochrome *c* oxidase subunit 4 (COXIV). As expected, in response to the overload stimuli, the plantaris muscles displayed a significant increase in COXIV protein content (Fig. 3a; Fig. S3a). Conversely, in response to the unload stimuli, the soleus muscles displayed a significant reduction in COXIV protein when compared to their sham controls (Fig. 3b; Fig. S3b).

### Alterations in CL content and 18:2*n*6 composition

**Figure 4:**
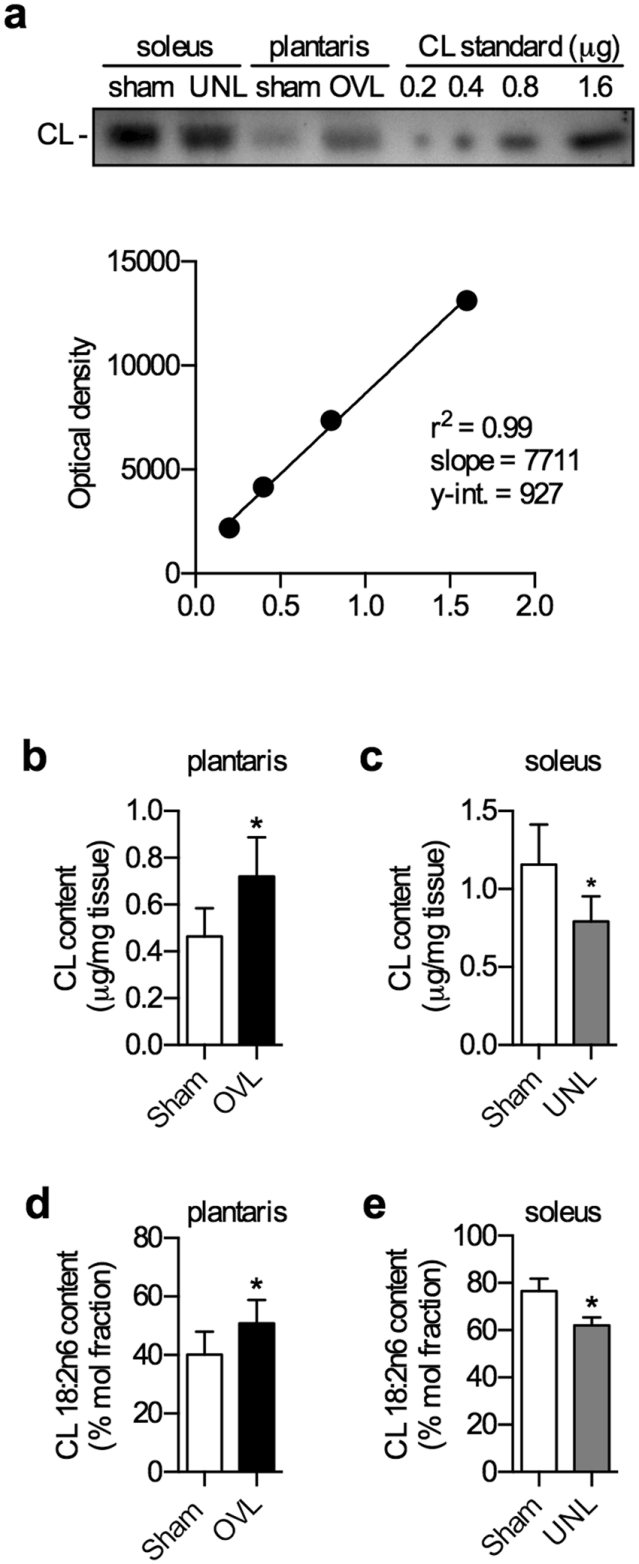
CL content and CL 18:2*n*6 composition are altered in the overloaded (OVL) plantaris and unloaded (UNL) soleus. (**a**) A representative HPTLC chromatogram used to generate a standard curve to quantify the absolute amounts of CL content. CL content is increased in the overloaded plantaris (**b**) and decreased in the unloaded soleus (**c**) muscles compared with their respective shams. CL 18:2*n*6 composition analyzed with gas-chromatography is significantly higher in the overloaded plantars (**d**) and trending to be lower in the unloaded soleus (**e**) compared with their respective shams. *p ≤ 0.05 using a paired t-test (two-tailed), n = 6 per group.

Using densitometric HPTLC, we quantified the absolute concentrations of CL (Fig. 4a). Our results show that sham soleus, on average, is more abundant (~2.5-fold) with CL compared with sham plantaris (*p* = 0.03). In response to the overload stimuli, plantaris displayed a ~1.5-fold increase in CL content (Fig. 4b), whereas unloaded soleus exhibited a 32% decrease (Fig. 4c). With respect to CL 18:2*n*6 composition, sham soleus CL are more enriched (+30%) with 18:2*n*6 compared to sham plantaris (*p* = 0.01). In response to the overload stimuli, plantaris displayed a significant 10% increase in CL 18:2*n*6 composition (Fig. 4d). The CL 18:2*n*6 composition was approximately 10% lower in unloaded soleus compared with sham however, this was not significant (*p* = 0.10, Fig. 4e).

### Taz protein expression in response to overload and unload stimuli

**Figure 5:**
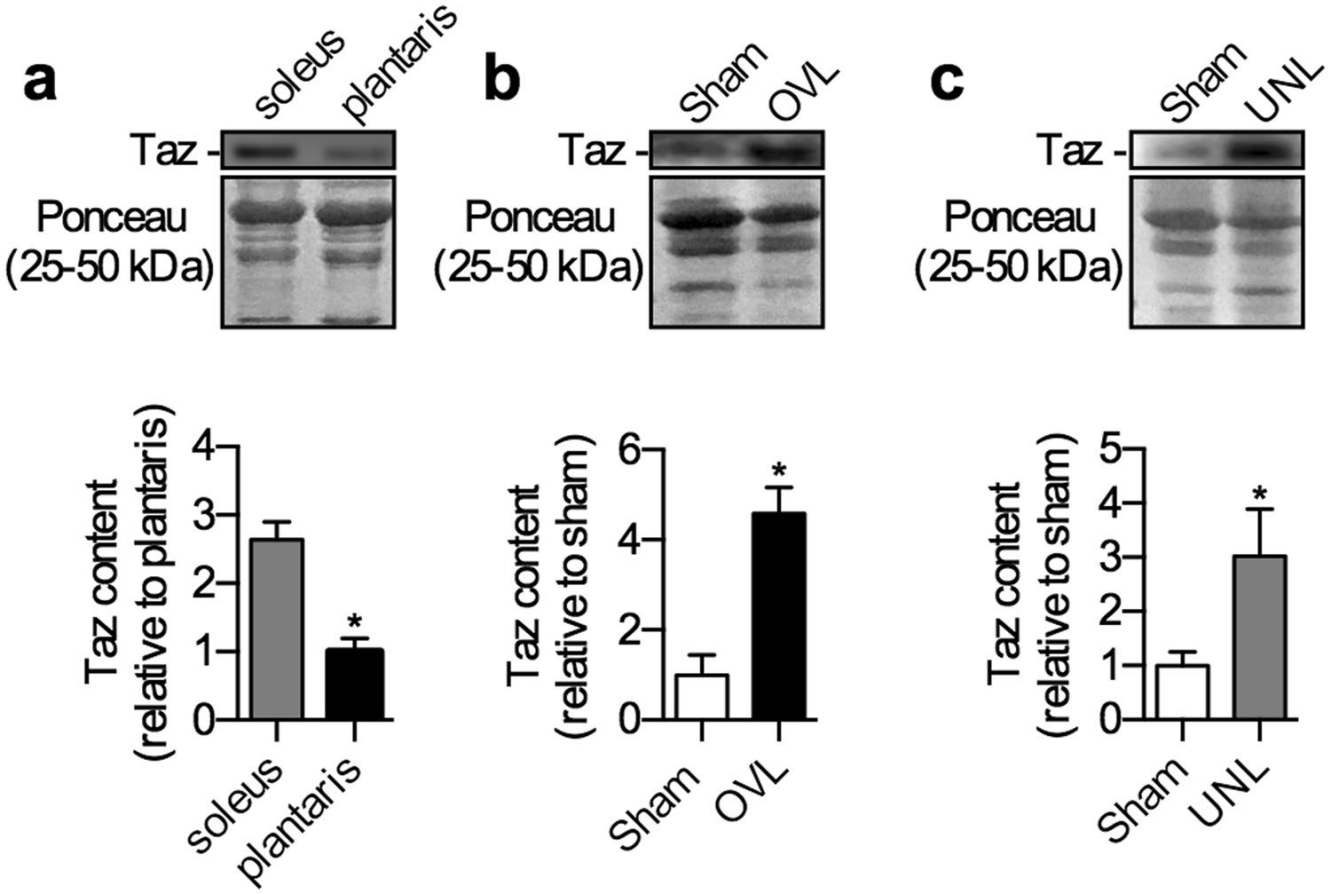
Taz protein expression is higher in the soleus compared with plantaris and is upregulated in response to both overload (OVL) and unload (UNL) stimuli. (**a**) Taz protein expression (~27 kDa) in the sham plantaris and soleus muscles. (**b**) Taz protein in the OVL plantaris compared with sham. (**c**) Taz protein in the UNL soleus compared with sham. *p ≤ 0.05 using a Student’s t-test (**a**, two-tailed) or paired t-test (**b** and **c**, two-tailed), n = 6 per group.  Please refer to Fig. S4 for full length Western blots.

As with CL content and CL 18:2*n*6 composition, Taz protein was higher in sham soleus compared with sham plantaris (+2.6-fold, Fig. 5a; Fig. S4a). In plantaris, our results revealed a 4.5-fold increased Taz protein content in response to the overload stimuli compared with sham (Fig. 5b; Fig. S4b). A similar observation was made with soleus, which displayed a 3.0-fold increase in Taz protein content in response to the unload stimuli compared with sham (Fig. 5c; Fig. S4c).

## Discussion

In the present study we sought to characterize the dynamic nature of CL content, CL 18:2*n*6 composition and Taz protein content in response to muscle overloading and unloading stimuli. Specifically, we tested the hypothesis that along with the shift towards the slow-oxidative phenotype, which have greater mitochondrial content, an increase in CL content, CL 18:2*n*6 composition, and Taz protein would be observed in overloaded plantaris compared with sham. Conversely, in unloaded soleus induced by tenotomy, we hypothesized that the slow-to-fast fiber type shift, which have lower mitochondrial content, would correspond with a reduction in CL content, CL 18:2*n*6 composition, and Taz protein. To our knowledge, this is the first study examining these effects in response to muscle overloading and unloading stimuli.

Skeletal muscle is a heterogeneous tissue comprised of several different fiber types, which can be classified into groups based on contractile speed, myosin heavy chain (MHC) expression, and metabolic capacity^18^. There are 4 major MHC isoforms that can be expressed in adult mammalian skeletal muscle and that can be used to identify the different fiber types, with one slow isoform (MHCI) and three fast isoforms (MHCIIa, MHCIIx and MHCIIb)^18, 20^. With respect to metabolic capacity, type I and IIA fibers exhibit the greatest oxidative potential and mitochondrial content, whereas type IIX and IIB fibers are more glycolytic and are less abundant with mitochondria^18, 20, 27^. Plantaris is a fast-twitch glycolytic muscle that, in the C57BL/6 mouse, is dominated by type IIB fibers (~46%), followed by type IIX (~22%) and type IIA (~20%), with virtually no type I^20^. Conversely, soleus from the C57BL/6 mouse contains a large proportion of type I and IIA fibers (30% and 50%, respectively) with relatively less type IIX (11%) and a very low amount of type IIB (3%)^20^. Therefore, it is not surprising that we observed greater CL content and CL 18:2*n*6 composition in sham soleus compared with sham plantaris, since these parameters are associated with oxidative capacity and mitochondrial content^5–7, 11, 28^. In fact, our results are in line with those from a previous study, where comparisons between cardiac muscle and extensor digitorum longus, which like plantaris is a fast-twitch glycolytic muscle enriched with type IIB fibers^20^, revealed greater CL content and CL 18:2*n*6 composition in cardiac muscles compared with EDL^11^.

However, these results are inconsistent with our previous findings in rat skeletal muscle, where we found that CL 18:2*n*6 composition was significantly greater in plantaris compared with soleus^4^. This apparent discrepancy may be explained by fiber type differences that occur between mouse and rat skeletal muscles, whereby plantaris from rat contain less type IIB fibers (~16%) and more type IIX fibers (~45%); and rat soleus is almost completely comprised of type I fibers (97%) with only 3% type IIA fibers^20^. Although the type IIX fibers exhibit relatively high glycolytic potential compared with type I and IIA fibers, the type IIX fibers in rodents also display considerable succinate dehydrogenase activity, suggesting that these fibers have an appreciable amount of oxidative potential^20^. Moreover, in rodents, type IIA fibers display greater oxidative potential when compared to type I fibers^20^. Altogether, the combined effects of lower type IIA and type IIB content in rat soleus and plantaris, respectively, may contribute to the divergent results seen here in mice with regards to CL 18:2*n*6 composition. Nevertheless, the results of the present study are consistent with the notion that CL content and CL 18:2*n*6 composition follows mitochondrial content^5–7, 11, 28^.

In response to tenotomizing soleus and gastrocnemius tendons, the functionally overloaded plantaris underwent hypertrophy and transition towards a slow-oxidative phenotype after a 2 week-period, similar to what has been seen previously^21–24^. Specifically, plantaris:body weight ratios were significantly increased; and with Western blotting, we were able to demonstrate a significant reduction in MHCIIb and significant increases in MHCIIx, IIa, and I, indicative of a transition towards a slow-oxidative phenotype. We also observed a significant increase in COXIV protein, which is supportive of increased mitochondrial content in the overloaded plantaris compared to sham. Consistent with this increase in mitochondrial content and consistent with our hypothesis, we found a significant increase in CL content, CL 18:2*n*6 composition and Taz expression in the overloaded plantaris compared with sham. Even in the basal state, we found that Taz protein is higher in slow-oxidative muscles (sham soleus) compared with fast-glycolytic muscles (sham plantaris). To our knowledge, this is the first study to demonstrate muscle type differences in Taz protein. Overall, our results are partly consistent with a previous study whereby chronic muscle use led to increases in CL content; however, CL 18:2*n*6 composition was not examined^29^. Furthermore, in the study by Ostojic *et al*., chronic contractile activity did not lead to any changes in Taz expression, however, only mRNA levels were examined, and the fact that mRNA and protein content are not directly proportional is generally well accepted.

By tenotomizing soleus and gastrocnemius, we were able to also study soleus as a model of muscle unloading. In response to unloading, soleus underwent significant atrophy and a fiber type transition towards a fast-glycolytic phenotype after 2 weeks, as seen previously^25, 26^. Specifically we observed a significant reduction in soleus:body weight ratio and a slow-to-fast fiber type transition with a significant decrease in MHCI expression and significant increases in MHCIIa and IIx. In addition, we found a significant reduction in COXIV protein content, which supports an overall reduction in mitochondrial content. Corresponding with these changes in fibre type makeup and mitochondrial content, we observed a significant reduction in CL content; and although CL 18:2*n*6 composition was lower in unloaded soleus this did not reach significance (*p* = 0.10). This was likely due to 1 of the 5 pairs analyzed (sham vs unload) not displaying a reduction in CL 18:2*n*6 composition in response to the unload stimuli. Removal of this pair draws the *P* value towards significance (*p* = 0.01). Strikingly, we also found a significant increase in Taz protein in the unloaded soleus compared with sham, which was inconsistent with our original hypothesis. Although purely speculative, we view that this increase in Taz protein may be an adaptive response that acts to combat the reductions in mitochondrial CL content and CL 18:2*n*6 composition. Altogether, these findings in the unloaded soleus are partly consistent with a previous study whereby muscle disuse led to a decrease in CL content; however, CL 18:2*n*6 composition was not examined^29^. Furthermore, in the study by Ostojic *et al*., muscle disuse via denervation did not lead to any changes in tafazzin expression, however, only mRNA levels were examined.

Collectively, our findings demonstrate the dynamic nature of CL content, CL 18:2*n*6 composition and tafazzin protein, which for the most part, follows the slow oxidative phenotype and increased mitochondrial content. Indeed, CL content itself is thought to be one of the best markers of mitochondrial content in skeletal muscle^2^. In the overloaded plantaris, increased mitochondrial content supports the increased contractile metabolic demand of becoming a postural muscle in the absence of functional soleus and gastrocnemius. Conversely, unloading a soleus muscle reduces its metabolic demand, which likely contributes to the reductions in mitochondrial content. Importantly, in conditions of muscle unloading and/or chronic muscle inactivity, there is a significant production of ROS leading to detrimental processes such as cell death, proteolysis, and inhibition of protein synthesis ultimately causing muscle atrophy^26, 30–33^. ROS-mediated mitochondrial dysfunction triggers a defense-oriented response to remove aberrant mitochondria from the cell via mitophagy^34, 35^. Mitophagy may act reduce the overall mitochondrial content in unloaded soleus and could also explain the reductions in CL content and CL 18:2*n*6. Furthermore, 18:2*n*6 is a highly peroxidizable fatty acid, and thus, the apparent reductions seen here in response to soleus unloading could be due, in part, to increased lipid peroxidation. Alternatively, it is well-established that reductions in CL content and CL 18:2*n*6 composition leads to structural and functional mitochondrial defects, which enhances mitochondrial ROS production as seen in patients with Barth syndrome^7, 36, 37^. Thus, it is also possible that reductions in CL content and CL 18:2*n*6 composition seen in unloaded soleus could pathologically contribute to ROS production and muscle atrophy, and further strengthens our view of an increase in Taz protein representing an adaptive response in this model.

This highlights the notion that CL content and CL 18:2*n*6 composition may be more than just biomarkers of mitochondrial content. It is well known that these parameters are associated with increased mitochondrial function and respiration. For example, CL is known to induce membrane curvature in the inner mitochondrial membrane, which is important for generating the characteristic folds within the mitochondrial cristae that increase the surface area in which the mitochondrial respiratory complexes reside^38^. Indeed, recent results in human vastus lateralis muscle reveals that mitochondrial cristae density is a critical factor in mitochondrial respiration and oxidative capacity^39^. In this respect, CL is also thought to function as glue that holds the respiratory complexes in tight association with one another^7, 38^. Thus, the increase in CL content in the overloaded plantaris may function to increase membrane curvature and enhance the association between the respiratory complexes thereby ultimately leading to augmented mitochondrial respiration. Conversely, the reduction in CL content in the unloaded soleus may lead to reductions in membrane curvature and a ‘loose’ association between the respiratory complexes ultimately lowering mitochondrial respiration. With respect to CL 18:2*n*6 composition, we and others have demonstrated the positive impact of CL 18:2*n*6 composition on mitochondrial enzymatic activity^12–14^. Thus, the increase in CL 18:2*n*6 composition in the overloaded plantaris may be necessary to support an increased mitochondrial content and meet the increased contractile metabolic demand of becoming a postural muscle in the absence of functional soleus and gastrocnemius. In contrast, the reduction in CL 18:2*n*6 in the unloaded soleus may be reflective of decreased energetic demands associated with muscle unloading^40^.

Indeed, we are limited in our study as we may only speculate that these changes in CL content and CL 18:2*n*6 composition can lead to changes in mitochondrial respiration in our models. However, it is important to note that reductions in CL content and CL 18:2*n*6 composition are well known to reduce mitochondrial respiration and induce ROS production in Barth syndrome^7, 36, 37^. Nevertheless, future studies, aimed at altering CL content and CL 18:2*n*6 composition while measuring mitochondrial respiration will provide further insight. Future studies could use dietary intervention as linoleic acid supplementation of Barth syndrome fibroblasts restored CL content and CL 18:2*n*6 composition and is considered to be a viable therapeutic strategy for this myopathy^41^. Furthermore, the results from our study and the results from previous studies on Barth synrome^7, 36, 37^ suggest that targeting tafazzin in both the overloaded plantaris and unloaded soleus may provide a feasible method to alter CL content and CL 18:2*n*6 composition. Our laboratory is currently generating a *tafazzin* knockdown mouse colony^11, 42^ to better understand the physiological role of increased Taz expression in the overloaded plantaris and unloaded soleus. In essence, it would be of interest to determine the effects of altering CL content and CL 18:2*n*6 composition not only on mitochondrial respiration, but also on the physiological adaptations (ie. muscle mass and oxidative/glycolytic phenotype) that occur in the overloaded plantaris and unloaded soleus.

In summary, we have characterized the changes that occur with CL content, CL 18:2*n*6 composition, and Taz protein in response to plantaris overloading and soleus unloading. We suspect that the changes in CL content and CL 18:2*n*6 composition are reflective of changes in fiber type composition and, more specifically, mitochondrial content. However, it is possible that these changes in CL content and CL 18:2*n*6 may act to alter mitochondrial function and respiration, and future studies are required to substantiate these claims. Nevertheless, our study highlights the dynamic nature of CL content, CL 18:2*n*6 composition and Taz expression in mouse skeletal muscle, thereby providing the necessary baseline characterization required to further elucidate the roles of CL content, CL 18:2*n*6, and Taz protein in overall skeletal muscle health.

## Methods

### Mice

Six adult (4–6 month) male C57BL/6 mice (29.1 ± 1.2 g) were used in this study. Animals were housed in an environmentally controlled room with a standard 12:12-hour light-dark cycle and allowed access to food and water *ad libitum*. All animal procedures were reviewed and approved by the Brock University Animal Care and Utilization Committee and carried out in accordance with the guidelines established by the Canadian Council on Animal Care.

### Simultaneous plantaris mechanical overload and soleus unload

To overload plantaris while unloading soleus, mice were first anaesthetized with 2% isoflurane in a precision vaporizer. Next, soleus and gastrocnemius tendons were transected as previously described^23^ and mice were left for two weeks in individually housed cages to adapt. Subsequently, the mice were anaesthetized in an induction chamber using isoflurane (5%), transitioned to a nose cone (5% isoflurane) and then placed on a surgical bed for muscles to be surgically removed. Plantaris (sham and overload) and soleus (sham and unload) were then homogenized in homogenizing buffer (250 mM sucrose, 5 mM HEPES, 0.2 mM PMSF, 0.2% [w/v] NaN_3_) in a 10:1 ratio and stored at −80 °C until further analysis.

### Cardiolipin concentration analysis with HPTLC

Total lipids from muscle homogenates (1.25 mg) were extracted as previously described^43^ and the lipid extract was spotted onto high-performance thin layer chromatography plates (HPTLC; 5633-5, EMD Chemicals, Darmstadt, Germany) using a chloroform:methanol:acetic acid:water (100:75:7:4) solvent system to separate individual phospholipids^4^. A standard curve (0.2, 0.4, 0.8, 1.6 μg) of bovine heart cardiolipin (C0563, Sigma Aldrich, MO, USA) was also loaded onto each HPTLC plate, and after allowing the solvent to run up each plate for 45 min, the plates were then charred at 180 °C with a 10% (w/v) copper sulfate in 8% phosphoric acid solution for 15 min^44^. Images of the HPTLC plates were captured using a CCD camera on a Fluorchem 5500 imaging station (Alpha Innotech, CA, USA) under reflective white light. Densitometry analyses were then performed using ImageJ (National Institutes of Health, MA, USA) and the standard curve of bovine heart cardiolipin was used to calculate the absolute CL concentration (per 1.25 mg of muscle) in plantaris and soleus.

### CL 18:2*n*6 composition

To determine the CL 18:2*n*6 composition, total lipids from 1.25 mg of muscle homogenates were extracted and then spotted onto a separate HPTLC plate and allowed to run in the same solvent and for the same time as discussed in the previous section. Next, the separated phospholipids on the HPTLC plates were developed by spraying a dichlorofluoroscein (DCF) solution (methanol:water [1:1], and 2′,7′-DCF filtered and washed with petroleum ether) onto the plate and setting it into a chamber containing 25% ammonium hydroxide for 5 min. The HPTLC plate was viewed under ultraviolet light and the CL bands were marked and scraped into individual 15 ml kimex culture tubes and allowed to methylate in 2 ml of 6% H_2_SO_4_ (w/v in methanol) overnight at 50 °C. Fatty acyl methyl esters were extracted with petroleum ether, dried down, reconstituted into dichloromethane (20 μl), and injected (2 μl) into a gas chromatograph (Trace GC Ultra, Thermo Electron, Milan, Italy) fitted with a split/splitless injector, a fast flame ionization detector, and Triplus AS autosampler (Trace GC Ultra, Thermo Electron) as previously described^45^. CL fatty acyl methyl esters were separated on an UFM RTX-WAX analytical column (Thermo Electron) using helium as a carrier gas. Fatty acids were identified by comparison of retention times with those of a known standard (Supelco 37 component FAME mix, Supelco, PA, USA), and absolute amounts (nmol) were calculated with the aid of the internal standard, tridecanoic acid (13:0), which was added to the samples immediately prior to the methylation process. The percent mol fraction of 18:2*n*6 was calculated using the sum of the absolute amounts of the individual fatty acyl methyl esters as the denominator.

### Western blotting

Western blotting was performed to examine the protein expression of MHCI, MHCIIa, MHCIIx, MHCIIb, COXIV, and Taz as previously described^46^. Briefly, proteins from muscle homogenates (5–10 μg for MHC; and 30–60 μg for Taz) were solubilized using Laemmli buffer^47^, and then electrophoretically separated using standard glycine-based SDS-PAGE (7.5% total acrylamide for MHC; and 13% total acrylamide for Taz). Separated proteins were then transferred onto 0.2 μm polyvinylidene difluoride (PVDF) membranes (Immuno-Blot, BioRad, CA, USA) using a semi-dry transfer setting on the Trans-blot Turbo Transfer System (BioRad). Next, membranes were probed with primary antibodies directed against MHCI (BA-F8, 1:100, Developmental Studies Hybridoma Bank [DHSB]), MHCIIa (SC-71, 1:100, DHSB), MHCIIx (6H1, 1:100, DHSB), MHCIIb (BF-F3, 1:100, DHSB), COXIV (ab16056, 1:1000, Abcam), or Taz (1B10, 1:500, Novus Biologicals, CO, USA) diluted in 5% (w/v) milk in tris-buffered saline tween. Following this, membranes were immunoprobed with a goat-anti mouse horseradish peroxidase conjugated secondary antibody (sc-2005, 1:2000, Santa Cruz, TX, USA), and the antigen-antibody complexes were detected with Luminata Forte^TM^ (Millipore, MA, USA) or ECL Western Blot Substrate (BioVision, MA, USA) using a C-Digit^®^ Blot Scanner (LI-COR, NE, USA). Optical densities were analyzed using Image Studio^TM^ (LI-COR).

### Statistics

All values presented here are means ± standard error (SE). Comparisons for sham vs. overload or sham vs. unload were done using a paired t-test. A Student’s t-test was used to compare the levels of CL content, CL 18:2*n*6, and Taz protein in sham plantaris vs. sham soleus. Statistical significance was set to *p* ≤ 0.05.

## Electronic supplementary material

Supplementary information
