# Supplementary material for: Cardiolipin content, linoleic acid composition, and tafazzin expression in response to skeletal muscle overload and unload stimuli
Source: Sci Rep. 2017 May 17;7:2060. doi: 10.1038/s41598-017-02089-1 (PMC5435726; doi:10.1038/s41598-017-02089-1)
Supplement: Supplementary file 1 — Supplementary information [file 41598_2017_2089_MOESM1_ESM.pdf]

SUPPLEMENTAL INFORMATION

Cardiolipin content, linoleic acid composition, and tafazzin expression in response to skeletal muscle overload and unload stimuli

Val A. Fajardo, John S. Mikhaeil, Cameron Leveille, Caitlin Saint, Paul J. LeBlanc*

Department of Health Sciences, Brock University, St. Catharines, Ontario, Canada

Running head: Cardiolipin remodeling in skeletal muscle

*Corresponding author:
Dr. Paul J. LeBlanc
Department of Health Sciences,
Brock University
St. Catharines, ON
N2L 3G1
905-688-5550 ext. 4216
pleblanc@brocku.ca

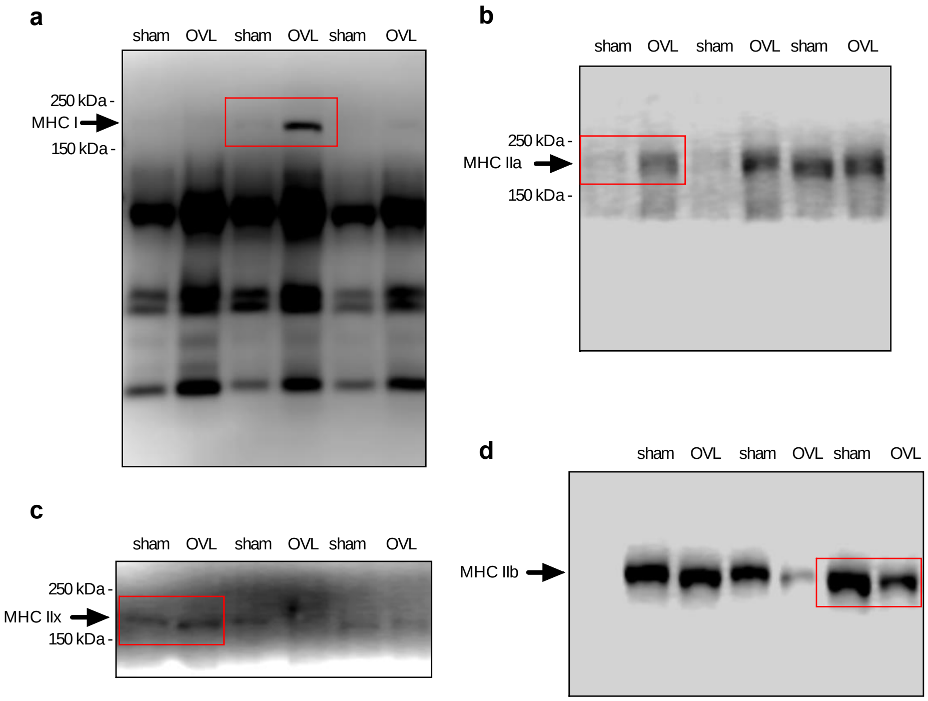

Supplemental Figure S1. Full-length Western blots for MHC I (a), MHC IIa (b), MHC IIx (c), and MHC IIb (d) from Figure 1. For **b-d** the PVDF membrane was cut to only incorporate proteins from >150 kDa.

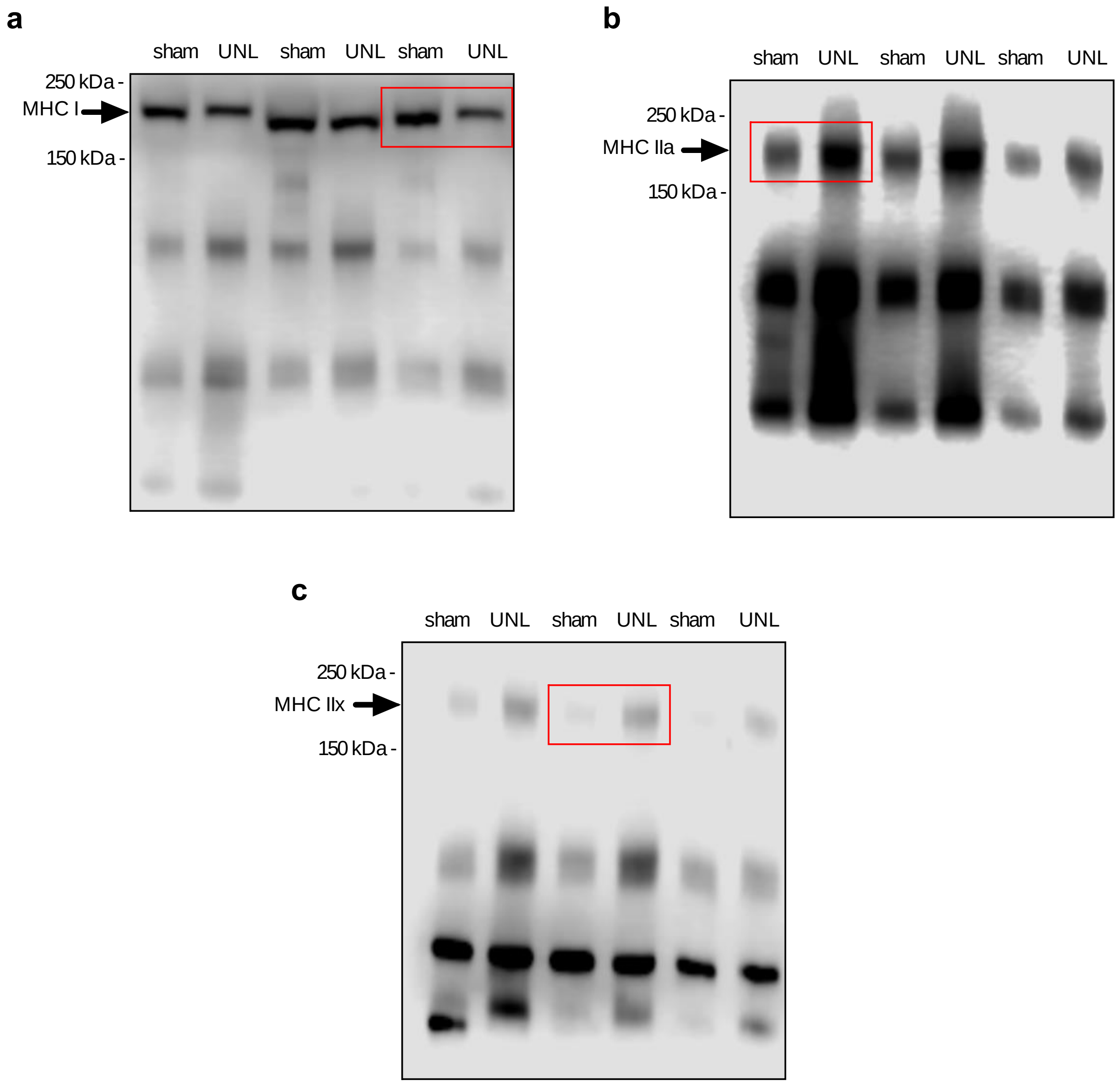

Supplemental Figure S2. Full-length Western blots for MHC I (a), MHC IIa (b), and MHC IIx (c) from Figure 2.

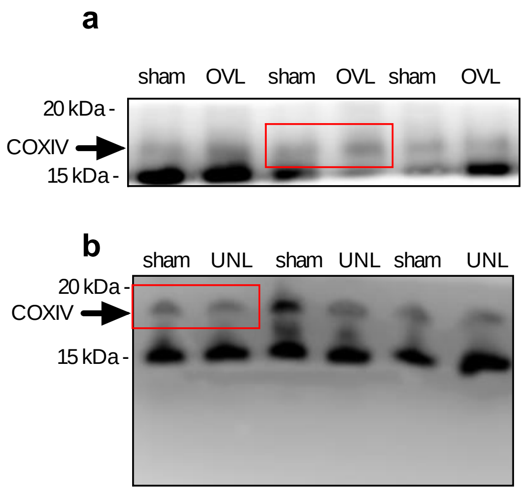

Supplemental Figure S3. Full length Western blots for COXIV in sham vs overloaded plantaris (a), and sham vs unloaded soleus (b) from Figure 3. For (**a**) the PVDF membrane was cut into a strip containing proteins from 15-20 kDa, whereas for (**b**) the PVDF membrane was cut to incorporate all proteins below 20 kDa. COXIV was recognized as a 17 kDa protein.

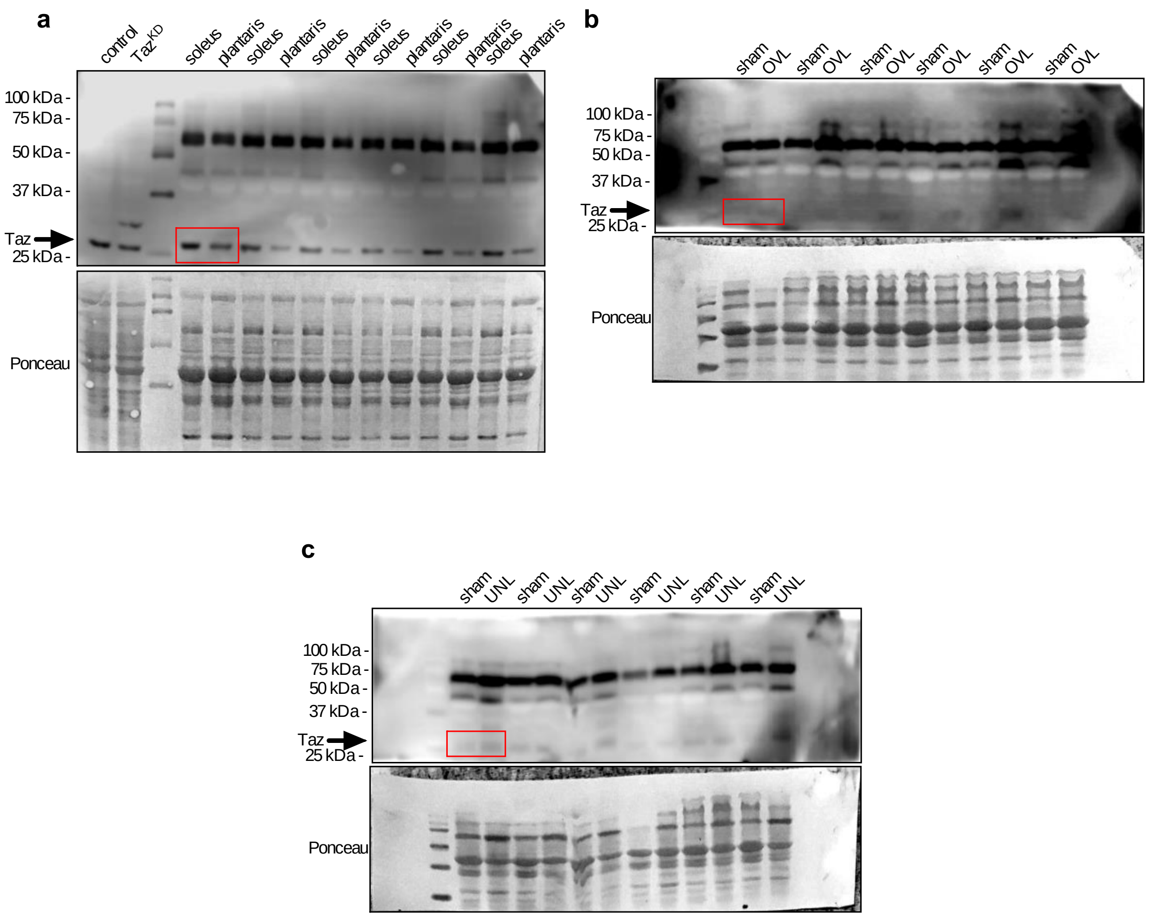

Supplemental Figure S4. Full-length Western blots for tafazzin (Taz) from Figure 5. For (**a**), the control and tafazzkin knockdown (Taz^{KD}) cells were a kind gift from Dr. Aaron Schimmer (University of Toronto). Taz was recognized as a 27 kDa protein.
